# Parents’ Social Status and Children’s Daily Physical Activity: The Role of Familial Socialization and Support

**DOI:** 10.1007/s10826-017-0808-3

**Published:** 2017-06-12

**Authors:** Michael Mutz, Peggy Albrecht

**Affiliations:** 1 Institute of Sport Science, Justus-Liebig-Universität Gießen, Gießen, Germany; 2 Faculty of Social Sciences, Georg-August-Universität Göttingen, Göttingen, Germany

**Keywords:** Exercise, Sport, Health, Family support, Social inequality, Childhood

## Abstract

Physical activity is a health relevant factor, particularly in affluent societies where overweight and obesity are increasingly prevalent, even among children. Understanding the development of physical activity patterns in childhood is thus an important issue for health promotion. Following socialization theory, this study describes and explains differences in objectively measured moderate-to-vigorous physical activity (MVPA) in a socially and ethnically mixed sample of 6- to 11-year-old children in Germany. MVPA levels were objectively measured with accelerometers over the course of six consecutive days (Wednesday to Monday). Parents’ attitudes and practices as well as the family’s socio-economic status (SES) were assessed from the parents via questionnaires. Results indicate that MVPA levels of children vary with gender, but not with age and ethnicity. Moreover, parental SES, parental support for the child’s sports activities, parents’ own sport activities and the parents’ belief in sports’ capacities to foster personality development, character building and social integration significantly predict the MVPA level of children. It is concluded that interventions to promote MVPA among children need to take family interactions and lifestyles into account and should address families in socio-economically underprivileged areas.

## Introduction

Physical activity is a health relevant factor, particularly in affluent societies, where sedentary lifestyles, overweight and obesity are increasingly prevalent among adults (Church et al. 2011; Flegal et al. 2002; Hallal et al. 2012; Ng et al. 2014) as well as children and youths (Fox 2004; Hallal et al. 2012; Ng et al. 2014; Wang and Lobstein 2006). Studies from Germany (Buksch et al. 2014; Lampert et al. 2007; Manz et al. 2014) as well as many other European countries (Riddoch et al. 2004; Verloigne et al. 2012) indicate that children’s average physical activity levels are rather low and often do not meet the minimum level of 60 min of moderate to vigorous physical activity (MVPA) per day, as recommended by the World Health Organization (WHO 2010). Therefore, the promotion of sport and exercise in all stages of the life-course has become a major pillar of health policies (Kahn et al. 2002; Heath et al. 2012; Sallis Bauman and Pratt 1998). Prior research has shown that high and continuous levels of physical activity in childhood and youth are likely to lead to physically active lifestyles in adulthood (Telama et al. 2005). It seems likely though, that sedentary lifestyles are also habitualized in childhood which can negatively affect cardiovascular risk factors in later life stages (Healy et al. 2011; Katzmarzyk et al. 2009; Qi et al. 2015). Hence, further understanding of the development of physical activity and inactivity patterns in childhood are of crucial importance (Hamilton et al. 2015).

Most previous studies have assessed activity levels of children either with activity diaries (Raudsepp 2006; Schoeppe and Trost 2015; Trost et al. 2003; Woll et al. 2011) or accelerometers (e.g., Aznar et al. 2011; Bringolf-Isler et al. 2009; Griffiths et al. 2013, 2016; Riddoch et al. 2004; Steele et al. 2010; Trost et al. 2002). The latter studies have often been concerned merely with a precise description of activity levels. Besides the finding that many children do not meet the activity guidelines, it was shown that the proportion of children below the recommended levels of MVPA is particularly large among girls (Aznar et al. 2011; Crespo et al. 2013; Griffiths et al. 2013; Kettner et al. 2013) and children from lower social status groups (Vorwerg et al. 2013). Moreover, MVPA levels drop with age (Trost et al. 2002; Riddoch et al. 2004). Studies based on activity diaries and questionnaires also have concurrently shown that children and youths from upper and middle class are more likely to be involved in sport and exercise (Jekauc et al. 2013; Mess and Woll 2012; Raudsepp 2006; Schoeppe and Trost 2015). Children’s sports and exercise participation is closely associated with their family’s socio-economic status (SES) and their parents’ lifestyle and support (Burrmann 2005; Edwardson and Gorely 2010; Loprinzi and Trost 2010; Nagel and Ehnold 2007; Wijtzes et al. 2014). Parents’ SES is not only correlated with children’s sports activities in leisure time and their membership in sports clubs, but also with participation in toddler gymnastics (Schmiade and Mutz 2012), motor abilities (Klein et al. 2011), swimming skills (Kuntz et al. 2016), the use of public swimming pools (Audrey et al. 2012) and outdoor play activity (Wijtzes et al. 2014).

However, the mechanisms and transmission paths that lead from parents’ SES to children’s physical activities are not nearly as well understood. Socialization theories generally state that behavior patterns and personality traits are developed in a life-long process and in interaction with the social and material environment. For example, Hurrelmann (2009) has described individuals as “productive processors of reality” and thus claimed that personality is not solely coined by environmental factors. Instead, subjects have agency to actively shape and co-construct their own development. However, when it comes to children it is also undoubted that the family is the primary entity for socialization and provides important stimuli that profoundly influence attitudes, values, cognition and behavior and thus partially predetermine children’s further development (Duncan et al. 2005). Hence, through family interactions attitudes and behavior patterns can be transmitted from the parent generation to their offspring.

This inner-familial socialization and transmission process can be applied to sport and exercise as well. Prior research suggests that several key mechanisms may be at work which all are compatible to the general socialization framework. It is typically claimed (a) that parents work as *role models* for their children, set an example and thus convey their own class-based sports orientations and practices to their children (Edwardson and Gorely 2010; Pugliese and Tinsley 2007). Hence, parents own sports activities are considered a crucial factor in the sports-related socialization process of their children. (b) Moreover, parents can provide social *support for sports activities* of their children actively, for instance, they could play sport together with their children, encourage them to start sports activities or accompany them to competitions (Burrmann 2005; Loprinzi and Trost 2010; Schoeppe and Trost 2015). (c) Besides active support from parents, sports activities are also fostered through *sports facilities in the home environment*. In families where children have access to a large variety of sports equipment, like skateboards, balls, bicycles, skiing equipment, gym and outdoor fitness equipment, it seems more likely that children are inspired to play sport in their leisure time (De Lepeleere De Bourdeaudhuij et al. 2015). (d) Finally, parent’s *attitudes and orientations* towards sports are considered of importance, for instance, the value they see in sports activities in childhood (Kahn et al. 2008; Hamilton et al. 2015). Some parents may believe sport to be extremely important in regard to personality development, social skills acquisition and character building, while others may be more skeptical in regard to these outcomes. In families where sport is given more value with regard to developmental outcomes, children may receive more stimulation and may be inclined to exercise by themselves (Wheeler 2011).

Children from higher social classes may be privileged with regard to all of these conditions of sports-related socialization. In general, their parents are more likely to be actively involved in sport by themselves (Breuer and Wicker 2008; Studer et al. 2011) and may associate sport with better developmental outcomes (Cachay and Thiel 1998). Moreover, they may be more likely to support their children’s sports activities, be it through active support or through investments into equipment (Burrmann 2005; Loprinzi and Trost 2010; Schmiade and Mutz 2012). Hence, the transmission of class-based inequalities from parents to children is likely to operate on these four mechanisms.

This paper aims to better understand the inner-familial transmission process through which social inequalities translate into differences in MVPA levels in children. We analyze this process in the age group of 6- to 11-year old children, an age in which the course is likely to be set for later life-stages due to a habitualization of sport and exercise patterns. In this study physical activity measures derived from the children and questionnaire data collected from the parents are combined. Physical activities are recorded with accelerometers, a technology which allows to assess objectively the duration and intensity of physical activities performed by the children. This measure thus includes MVPA beyond the narrow scope of organized sports and physical education, for instance, walking, playing at playgrounds or romping around. Individual differences in daily levels of physical activity are then explained by a multitude of possible socialization factors, collected with parent questionnaires. This allows to identify the most important ‘transmission belts’ that lead from parents’ attitudes, values and behaviors to their offspring’s daily physical activity routines. Based on the results of previous research it is hypothesized that MVPA in children increases with (1) a higher educational level of the parents; (2) a higher income level of the family; (3) the support the child receives from parents for sports activities; (4) the sports activities the parents pursue for themselves; (5) the parents’ belief in sport-for-good, i.e. in sports’ capacity to positively impact personality and development; (6) the sports equipment available in the family’s home.

## Method

### Participants

Participants were 150 pupils in elementary schools together with their parents. All families lived in Göttingen, Germany, a medium size university town with 116,000 inhabitants. The families participated voluntarily in this study and were contacted via schools. For this purpose, three elementary schools were selected, which all are characterized through a socially and ethnically mixed composition of the student body. The children in the final sample were in the age range between 6 and 11 years (*M* = 8.23, SD = 1.24, *Min* = 6, *Max* = 11). The sample consists of 80 boys (53%) and 70 girls (47%). Various socio-economic strata (according to income and educational levels) as well as ethnic backgrounds are represented. For instance, 35% of the children have a migration background, that is, either the child or the parents are born in a foreign country. A detailed sample description is given in Table 1.

**Table 1:** Sample description

Sample description					
	Mean	Median	SD	Min	Max
MVPA	73.2	71.9	25.8	18.9	145.8
VPA	23.5	22.7	13.6	2.4	71.3
MPA	49.7	49.4	14.4	14.9	84.0
Child’s gender^a^	0.47	–	–	0.0	1.0
Child’s age	8.23	8.00	1.24	6.0	11.0
Child overweight/obese^b^	0.11	–	–	0.0	1.0
Immigrant background^c^	0.35	–	–	0.0	1.0
Religious affiliation^d^	0.84	–	–	0.0	1.0
Mean daily temperature	9.23	8.70	5.56	−2.24	23.9
No. of children	2.16	2.00	0.84	1.0	5.0
Parents age	40.2	40.5	6.07	26.0	55.0
Parents net income	4.47	4.50	2.19	1.0	8.0
Parents education	3.79	4.00	1.12	1.0	5.0
Parents sports activity	3.01	3.00	1.18	1.0	5.0
Parents support for sport	3.23	3.25	0.62	1.75	4.5
Parents belief in sport-for-good	4.50	4.60	0.45	2.8	5.0
Sports equipment at home	9.11	9.00	2.89	1.0	16.0

^a^ 1 = female (47%), 0 = male (53%)

^b^ 1 = overweight/obese (11%), 0 = not overweight (89%)

^c^ 1 = either the child or the parents are born in a foreign country (35%), 0 = child and parents are born in Germany (65%)

^d^ 1 = at least one parent has a religious affiliation (84%), 0 = both parents are unaffiliated with any religion (16%)

### Procedures

At the core of this research are effects of family SES and support on objectively-measured physical activity levels in children. Data collection took place between June 2015 and May 2016. Except from summer breaks and Christmas holidays, data was collected in four families each week. Children wore a triaxial accelerometer (ActiGraph GT3X+) for 6 consecutive days (Wednesday to Monday) on an elastic belt over the hip. Wearing time was defined as wake time and thus started in the morning directly after awakening and ended when the child went to bed in the evening. At one occasion during the week, the parents were asked to fill out a questionnaire addressing their socio-economic and socio-cultural resources as well as parental support for the child’s activities. During the whole week every family was supervised by a research assistant, who explained the functioning and carrying method of the accelerometer, adjusted the device to the child’s body proportion, so that convenience while wearing was assured, and inquired about wearing compliance. At the end of the data collection a compensation of 80 € was disbursed to the family.

### Measures

#### Objective physical activity

Objective physical activity was collected via accelerometer. Activity data were stored at 10-s intervals. Non-wear-time, defined as at least 10 min with consecutive counts of zero activity, was removed from the summation of overall wear-time. Two children whose wear time did not reach at least 480 min per day for at least three weekdays and one weekend day were excluded from the analyses. However, for 148 children the activity scores were calculated based on all days with a wearing time above 480 min, usually 5 or 6 days. As long as mean scores are based on at least 4 days, they are considered valid measures for ‘general’ physical activity (Gabrys et al. 2015). Time spent in moderate and vigorous physical activity (MPA, VPA) was extracted, using the recommended thresholds reported by Evenson et al. (2008). Thus, intensity thresholds applied were 2296 to 4011 counts per minute for MPA and >4012 counts for VPA.

#### Education

Parents could indicate their highest educational degree accomplished, from (1) ‘no degree’, (2) ‘lower secondary level’ (9 years of school attendance), (3) ‘medium secondary level’ (10 years of school attendance), (4) ‘upper secondary level’ (12 to 13 years of school attendance) to (5) ‘tertiary level’ (including all university degrees). The mean score of both parents is used.

#### Net income

In one parent questionnaire the net household income was inquired. Eight answer categories were provided, ranging from (1) <1000 €, (2) 1000–1500 €, (3) 1500–2000 €, (4) 2000–2500 €, (5) 2500–3000 €, (6) 3000–4000 €, (7) 4000–5000 € to (8) >5000 €. Income classifications are widely used in German survey research, e.g. the German General Social Surveys (www.gesis.org/en/allbus).

*Parents sports activities* were assessed via questionnaire. Parents were both asked to indicate how long they exercised in the last week. Answer categories include (1) ‘less than 30 min’, (2) ‘30 to 60 min’, (3) ‘1 to 2 h’, (4) ‘2 to 4 h’ and (5) ‘more than 4 h’. The mean score of both parents’ exercise duration was calculated. Similar measures which focus on the last week and ask for a summation of sports activities in hours and minutes have been widely used (for a review: Sylvia et al. 2014). However, previous studies often start with ‘<60 min’ (Karageorghis et al. 2005). To be better able to differentiate activity levels among less active individuals, we started with ‘<30 min’.

*Parents support for sports activities* was assessed with 4 items, which refer to instrumental support for sport activities, namely: ‘I exercise together with my child’; ‘I watch my child doing sport’; ‘I talk with my child about sport’; and ‘I drive my child to his/her sports activities’. Similar items have been used by Trost et al. (2003). Answer categories ranged from (1) ‘never’ to (5) ‘very often’. The final scale represents the mean of both parents’ answers. The scale has a unidimensional structure with all four items representing the same underlying factor. The reliability of the scale is acceptable (α = .75).

*Parents belief in sport for good* was measured with 5 items that referred to the belief in sports’ capacity to promote ‘character building and personality development’; ‘a positive body image’; ‘fairness and cooperation’; ‘social integration’; and ‘fitness and health’ among children. Both parents indicated their (dis)agreement with these statements on a 5-point Likert scale with (1) ‘totally disagree’ and (5) ‘totally agree’. The final ‘Sport-for-Good’ scale was calculated as the mean of both parents’ attitudes. The scale has a unidimensional structure, too, and its reliability can be considered acceptable (α = .74). Similar items have been used, for instance, in the 2007 International Social Survey Programme Module on ‘Leisure Time and Sport’ (ISSP Research Group 2009).

*Sports equipment in the home environment* was measured with an index of sports equipment available to the child at home. Parents were asked if their child has or can make use of a football, a basketball, a volleyball, a bicycle, diving goggles, a cross trainer, gym shoes, a sleigh, weights, a trampoline, skis, a Frisbee, a skateboard, roller skates, ice skates, a tennis or a badminton racket. The index was computed as the sum of all devices mentioned.

The four measures for sports-related socialization practices in the family, namely parents’ sports activities, parental support, parent’s beliefs in sport and the sports equipment at home, are only weakly correlated with Pearson correlations (*r*) between .07 and .26 (Table 2). Hence, they measure distinct aspects of the socialization process and it can be assumed that each aspect can potentially influence physical activity patterns of children independently.

**Table 2:** Correlations between measures for sports-related socialization in the family

Pearson correlations				
	(1)	(2)	(3)	(4)
(1) Parents’ sport activities	–			
(2) Support for sport activities	.13	–		
(3) Belief in sport-for-good	.15	.26**	–	
(4) Sports equipment at home	.07	.17*	.11	–

*p < .05; **p < .01

#### Controls

Regression weights for the above mentioned substantial variables are only estimated correctly, if confounding factors which are known to impact MVPA are controlled for. To control for characteristics of children, gender, age and weight status were considered as covariates. Weight status is used as a dummy variable, based on the child’s Body Mass Index, and separates overweight and obese children whose BMI is above the 90th percentile, according to national references (Robert-Koch-Institut 2013), from the rest. Furthermore, controls for the number of children in the household, the family’s religious affiliation and for an immigrant background were also included into the regression models. Previous studies from Germany (Burrmann et al. 2015) have shown that these factors determine children’s membership in sports clubs, so that it is likely that MVPA levels are also affected. Additionally, the parents’ age was included as it seems likely that younger parents are more active by themselves and play sport together with their children more often. The models also include a measure for mean daily temperature, as previous studies have shown that children’s MVPA is likely to vary with outside weather conditions (Lewis et al. 2016).

### Data Analyses

In a first step we describe the distribution (*M*, SD) of MPA, VPA and MVPA in our sample and document the proportion of children who reach WHO recommendations for daily physical activity. In order to describe the level of social inequality in MVPA, bivariate associations between the household’s income and the parents’ educational level with children’s MVPA are shown in a second step. In a third step, stepwise multiple regression models are calculated which point to robust dependencies between different aspects of sport-related socialization in the family and children’s daily physical activity routines. In particular, these model may give further clues on the mechanisms and intergenerational transmission paths which lead from social inequality among families to different levels of MVPA among children.

## Results

### MVPA Levels in Children

To assess the level of objective physical activity in 6- to 11-year old children, mean values and standard deviations for objective MPA, VPA and MVPA are documented (Table 1). According to accelerometer data, children in this age group were physically active for 73.2 min per day, on average. This amount of MVPA can be separated into 49.7 min of MPA and 23.5 min of VPA per day. The WHO recommendation of 60 min of MVPA was accomplished by 34% of the children in at least 5 of the 6 days where physical activity was recorded. Individual mean values of MVPA range from 18.9 min per day for the least active child up to 145.8 min for the most active child, thus indicating huge differences.

### Social Inequality of MVPA in Children

**Fig. 1:**
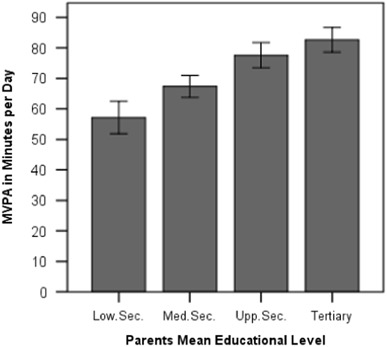
Children’s daily MVPA according to parent’s educational level. *Note:* One-way ANOVA. *F* = 6.23, η² = .118, *p* = .001. *Error bars* indicate the standard error of the mean

**Fig. 2:**
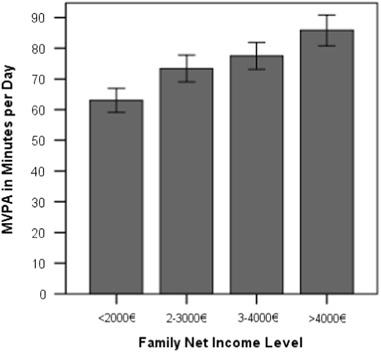
Children’s daily MVPA according to parent’s net income level. *Note:* One-way ANOVA. *F* = 4.94, η² = .102, *p* = .003. *Error bars* indicate the standard error of the mean

Individual differences in MVPA are moderately associated with family SES, as measured with parent’s educational level (*F* = 6.23, η² = .118, *p* = .001; see Fig. 1) and parent’s net income (*F* = 4.94, η² = .102, *p* = .003; see Fig. 2). Children whose parents have accomplished tertiary education reach mean MVPA levels of 82.6 min, whereas children with parents having lower secondary education achieve 57.2 min per day. Children from families with a net income above 4000 € per month reach 85.8 min of MVPA, whereas children from families with a household net income below 2000 € come at 63.0 min per day. The next section aims at explaining these differences.

### Predictors of MVPA in Children

A multiple regression analysis with MVPA as the dependent variable was run (Table 3). Variables were included in three steps into the model: first, the characteristics of the children (*model 1*); second, substantial and control variables for characteristics of the family (*model 2*); third, variables for parental sports-related lifestyle and support (*model 3*). Results for the child’s characteristics reveal a significant effect for gender. The gender effect is of remarkable size and indicates that girls spent 27.6 min per day less with MVPA compared to boys (*b* = −27.6; β = −.52; *p* < .01). Gender is the most important predictor in the regression model. Weight status and age are not significantly associated with MVPA levels.

**Table 3:** OLS Regression model for objective levels of MVPA in children

	MVPA I			MVPA II			MVPA III		
	*b*	β	*p*	*b*	β	*p*	*b*	β	*p*
Child characteristics									
Gender	**−27.6**	**−.52**	**<.01**	**−25.3**	**−.47**	**<.01**	**−24.2**	**−.45**	**<.01**
Age	**−**2.55	**−**.12	.128	**−**1.66	**−**.08	.323	**−**1.84	**−**.08	.249
Overweight/obesity	**−**6.62	**−**.08	.266	2.49	.03	.678	4.57	.06	.424
Parents SES*									
Parents’ net income	–	–	–	1.74	.14	.069	1.66	.13	.069
Parents’ education	–	–	–	**4.62**	**.19**	**.029**	**4.39**	**.18**	**.028**
Parents lifestyle and support*									
Parents’ sport activities	–	–	–	–	–	–	**2.72**	**.12**	**.045**
Support for sport activities	–	–	–	–	–	–	**5.63**	**.12**	**.042**
Belief in sport-for-good	–	–	–	–	–	–	**9.96**	**.17**	**.011**
Sports equipment at home	–	–	–	–	–	–	1.09	.11	.068
Controls									
Migrant background	–	–	–	0.16	.00	.969	2.45	.04	.524
Religious affiliation	–	–	–	**10.6**	**.16**	**.030**	**10.3**	**.15**	**.026**
No. of children	–	–	–	2.58	.08	.292	3.29	.10	.158
Parents’ age	–	–	–	**−**0.25	**−**.06	.465	**−**0.43	**−**.10	.207
Mean temperature	–	–	–	0.62	.13	.089	0.28	.06	.423
*R* ^2^		*0.30*			*0.43*			*0.52*	

*Note*: Ordinary least squares regression. *N* = 131. *As directions for these effects were hypothesized, significance is based on a one-tailed test. Bolded effects are significant with *p* < .05. *R* ^2^ values are significant with *p* < .001

Socio-economic status variables are introduced in the second step, together with controls for possibly confounding family characteristics. The multiple regression lends additional support that the parents’ education is associated with MVPA in children (*b* = 4.62; β = .19; *p* = .029). Financial resources have almost the same importance for MVPA, however the effect fails to reach conventional levels of significance (*b* = 1.74; β = .14; *p* = .069). Concerning the control variables, a religious affiliation of the parents has a positive effect on MVPA in children. Immigrant children accumulate similar levels of MVPA as native German children. For the number of children in the household and the parents’ age no significant results are revealed. The same applies to mean daily temperature, although the effect points into the expected direction that children accumulate more MVPA on warmer compared to colder days.

Finally, the measures for parental support for sport and exercise are introduced and still improve the fit of the model. Most notably, the parents’ belief in sport for good is significantly and strongly associated with MVPA in children (*b* = 9.96; β = .19; *p* = .011). Hence, MVPA in children increases particularly when parents believe in sports capacity to improve personality development and social integration. Moreover, the parents also seem to work as role models for their children. MVPA in children increases the more sport activities are pursued by the parents (*b* = 2.72; β = .12; *p* = .045). If both parents exercise “more than 4 h per week” the MVPA level of the child is estimated to be 10.9 min higher each day compared to a child whose parents both exercise “less than 30 min per week”. Third, parental support for sport activities of the child is also of relevance and significantly predicts MVPA in children (*b* = 5.63; β = .12; *p* = .042). A child who receives maximum support for sport and exercise from their parents (i.e. has a value of 5) is expected to spent 22.5 min more with MVPA compared to a child with minimum parental support (value of 1). The effect for the sports equipment available at home (*b* = 1.09; β = .11; *p* = .068) points into the expected direction but fails to reach significance.

The final model has a fit (*R*²) of .52 and suggests that individual differences in MVPA in children can be well explained and are largely a function of gender, parents’ education and religious affiliation, parents’ belief in sport as a means of character building and development, parents instrumental support of their child’s sports activities and of their own sports practices.

## Discussion

This study aimed at providing further insights into the transmission paths that lead from parents’ socio-economic status (SES) to children’s moderate-to-vigorous physical activity (MVPA). Whereas prior studies often focussed on organized settings for sport and exercise, this study was able to capture daily physical activity patterns, which include activities beyond the narrow scope of organised sports in clubs or schools. The findings demonstrated that daily patterns of MVPA in 6- to 11-year old children are subject to social inequalities and that parents’ SES—measured with educational and income levels—matters in this regard. However, more closely related to the child’s MVPA are the parents own sports activities, their support for the child’s physical activity—including their willingness to play sport together with their child—and their beliefs in sports’ capacity to foster personality development, social integration and character building. These aspects of sports-related socialization seem to play the key roles in the familial transmission process, indicating that attitudes, support and behavioural modelling represent distinct aspects of the familial sports-related socialization process. Although only marginally significant, the sports equipment at home also predicts MVPA in children. Hence, simply having a variety of sports equipment at home—e.g. balls, rackets, skates, boards—seems to motivate children to engage in more active leisure pursuits. A similar effect has been shown by De Lepeleere et al. (2015).

Gender was revealed as a strong predictor of MVPA in all model specifications, with girls being less active than boys. This effect is well-known (Verloigne et al. 2012). However, the present research demonstrates that the gender effect on MVPA is independent of parental SES and parental support. Moreover, neither age nor weight status were relevant predictors of MVPA. Regarding the effect of weight status, previous research has shown inconsistent results with some studies demonstrating that MVPA is inversely associated with overweight (Kahn et al. 2008), inversely associated with overweight in boys but not girls (Basterfield et al. 2014) or even positively associated with overweight (Kettner et al. 2013). Hence it seems that the association between weight status and MVPA in children is more complex than widely assumed and that overweight children are not per se physically inactive. Somewhat surprisingly, religious affiliation was revealed as a significant predictor of MVPA in children. Previous research has shown that religiousness is associated with participation in club-organized sports activities (Burrmann et al. 2015) and with spending less time with home-based entertainment activities, e.g. video games (López-Sintas et al. 2017). If such leisure patterns are associated with religion, they may produce higher levels of MVPA in religious children.

Generally, the results confirm findings of previous studies from Germany (Burrmann 2005; Nagel and Ehnold 2007; Schmiade and Mutz 2012) as well as from other countries (De Lepeleere et al. 2015; Hayoz et al. 2016; Raudsepp 2006; Schoeppe and Trost 2015), which have also addressed the intergenerational transmission of sport-related lifestyles in the family. These studies, however, have assessed sports activities by questionnaire and thus only in a narrower sense without including activity routines outside of sport. In line with the results of the present research, these studies demonstrated that parents’ sports activities and social support as well as their attitudes towards sport mediate the association between SES and children’s and adolescents’ organized sports activities. The present study has shown that these associations also hold true for daily physical activity routines, which include manifold activities outside the scope of organised sport, like playing at playgrounds, romping around, walking or cycling to school etc. Hence, social inequality is not only important for participation in club-organised sports, but also for general physical activity levels.

A very high share of children in Germany exercises in sports clubs. Membership figures reach its climax in the age group of 7- to 10-year olds, when roughly 70% of all children are sport club members (Manz et al. 2014). However, despite these high levels of participation in club-organized sporting activities, only a minority of the children meet the WHO physical activity recommendations, so that interventions in communities and schools are called for (Sutherland et al. 2016). Findings presented here suggest that interventions for physical activity promotion should aim at low status groups and possibly schools in underprivileged areas. Studies have shown that interventions to promote MVPA in schools can be effective in decoupling physical activity from SES (Vander Ploeg et al. 2014). Moreover, change processes in families could be initiated by increasing parents’ awareness for the health and developmental benefits of sport (Sanders and Burke 2014). However, future research is needed to provide evidence which of these measures are effective in fostering MVPA in children and disassociating MVPA levels from parents’ SES.

A strength of the present study is that MVPA levels have been measured objectively with accelerometers. This is particularly relevant in childhood as the main proportion of children’s physical activity takes place during unstructured play, (Bringolf-Isler et al. 2009) which is characterized through very short activity bouts (Ruch et al. 2013). These activities cannot be remembered reliably, especially not by children, and thus cannot be captured appropriately with diaries or questionnaires. A second problem of questionnaire-based data is that children and adolescents differ in their subjective understanding of physical activity. Some may subsume light activities like walking, cycling, or climbing stairs to MVPA while others only count vigorous sports activities. In view of these problems, objective measures can claim a higher validity compared to self-reported data (Beneke and Leithäuser 2008).

As a matter of course, this research is not free from limitations. First, the study has a cross-sectional design, so that children’s MVPA and familial practices were measured at the same time. Hence, no inferences on causality can be drawn from the data. Although there are good reasons to assume that parents influence MVPA in their children through their behaviour, support, and attitudes, it cannot be ruled out that a high level of MVPA in a child can also change parents’ stance towards sport and exercise. Second, this study was concerned with socialization processes in the family, and other social contexts of socialization (peers, schools etc.) were largely ignored. Finally, it has to be kept in mind that this study took place in a regional context and thus results may not be generalizable to the population of elementary school children in Germany. Although the sample represents various social and ethnic groups it has to be kept in mind that Göttingen is a University town with a high proportion of academics, so that findings may be biased due to idiosyncrasies of this particular region.

## References

[CR1] Audrey S, Wheeler BW, Mills J, Ben-Shlomo Y (2012). Health promotion and the social gradient: The free swimming initiative for children and young people in Bristol. Public Health.

[CR2] Aznar S, Naylor PJ, Silva P, Pérez M, Angulo T, Laguna M (2011). Patterns of physical activity in Spanish children: A descriptive pilot study. Child: Care, Health and Development.

[CR3] Basterfield L, Jones AR, Parkinson KN, Reilly J, Pearce MS, Reilly JJ, Adamson AJ (2014). Physical activity, diet and BMI in children aged 6-8 years: A cross-sectional analysis. BMJ Open.

[CR4] Beneke R, Leithäuser RM (2008). Körperliche Aktivität im Kindesalter – Messverfahren [Physical activity during childhood - methods of assessment]. Deutsche Zeitschrift für Sportmedizin.

[CR5] Breuer C, Wicker P (2008). Demographic and economic factors influencing inclusion in the German sport system – A microanalysis of the years 1985 to 2005. European Journal for Sport & Society.

[CR6] Bringolf-Isler B, Grize L, Mäder U, Ruch N, Sennhauser FH, Braun-Fahrländer C (2009). Assessment of intensity, prevalence and duration of everyday activities in Swiss school children: A cross-sectional analysis of accelerometer and diary data. International Journal of Behavioral Nutrition and Physical Activity.

[CR7] Buksch J, Inchley J, Hamrik Z, Finne E, Kolip P (2014). Trends in television time, non-gaming PC use and moderate-to-vigorous physical activity among German adolescents 2002–2010. BMC Public Health.

[CR8] Burrmann U (2005). Zur Vermittlung und intergenerationalen Vererbung von Sport(vereins) engagements in der Herkunftsfamilie [The influence and intergenerational heredity of sport(club) engagements from the nuclear family]. Sport und Gesellschaft – Sport and Society.

[CR9] Burrmann U, Mutz M, Zender U (2015). Jugend, Migration und Sport: Kulturelle Unterschiede und die Sozialisation zum Vereinssport [Youth, Migration and Sport: Cultural Differences and Sports-related Socialization].

[CR10] Cachay K, Thiel A, Hartmann-Tews I, Cachay K (1998). Kommerzialisierung und soziale Selektion im Kindersport [Commercialization and social selection in children’s sport]. Sport und soziale Ungleichheit [Sport and social inequality].

[CR11] Church TS, Thomas DM, Tudor-Locke C, Katzmarzyk PT, Earnest CP, Rodarte RQ, Martin CK, Blair SN, Bouchard C (2011). Trends over 5 decades in U.S. occupation-related physical activity and their associations with obesity. PLoS One.

[CR12] Crespo NC, Corder K, Marshall S, Norman GJ, Patrick K, Sallis JF (2013). An Examination of multilevel factors that may explain gender differences in children’s physical activity. Journal of Physical Activity and Health.

[CR13] De Lepeleere S, De Bourdeaudhuij I, Cardon G, Verloigne M (2015). Do specific parenting practices and related parental self-efficacy associate with physical activity and screen time among primary schoolchildren? A cross-sectional study in Belgium. BMJ Open.

[CR14] Duncan, G., Kalil, A., Mayer, S., Tepper, R., & Payne, M. (2005). The apple does not fall far from the tree. In Bowles S., Gintis H., & Groves M. (Eds.), *Unequal chances: Family background and economic success* (pp. 23–79). Princeton: Princeton University Press.

[CR15] Edwardson CL, Gorely T (2010). Parental influences on different types and intensities of physical activity in youth: A systematic review. Psychology of Sport and Exercise.

[CR16] Evenson KR, Catellier DJ, Gill K, Ondrak KS, McMurray RG (2008). Calibration of two objective measures of physical activity for children. Journal of Sports Sciences.

[CR17] Flegal KM, Carroll MD, Ogden CL, Johnson CL (2002). Prevalence and trends in obesity among US adults, 1999–2000. The Journal of the American Medical Association.

[CR18] Fox KR (2004). Childhood obesity and the role of physical activity. The Journal of the Royal Society for the Promotion of Health.

[CR19] Gabrys L, Thiel C, Tallner A, Wilms B, Müller C, Kahlert D (2015). Akzelerometrie zur Erfassung körperlicher Aktivität. Empfehlungen zur Methodik [Accelerometry for measuring physical activity. Recommendations on methods]. Sportwissenschaft.

[CR20] Griffiths LJ, Cortina-Borja M, Sera F, Pouliou T, Geraci M, Rich C (2013). How active are our children? Findings from the Millenium cohort study. BMJ Open.

[CR21] Griffiths LJ, Sera F, Cortina-Borja M, Law C, Ness A, Dezateux C (2016). Objectively measured physical activity and sedentary time: Cross-sectional and prospective associations with adiposity in the Millennium Cohort Study. BMJ Open.

[CR22] Hallal PC, Andersen LB, Bull FC, Guthold R, Haskell W, Ekelund U (2012). Global physical activity levels: Surveillance progress, pitfalls, and prospects. The Lancet.

[CR23] Hamilton K, Hatzis D, Kavanagh DJ, White KM (2015). Exploring parents’ beliefs about their young child’s physical activity and screen time behaviours. Journal of Child and Family Studies.

[CR24] Hayoz C, Klostermann C, Schlesinger T, Nagel S (2016). Zur Bedeutung sportbezogener Orientierungs- und Verhaltensmuster in der Familie für das Sportengagement Jugendlicher [The impact of sports behavior and orientations toward sports within the family on young people’s sports involvement]. Sport und Gesellschaft – Sport and Society.

[CR25] Healy GN, Matthews CE, Dunstan DW, Winkler EAH, Owen N (2011). Sedentary time and cardio-metabolic biomarkers in US adults: NHANES 2003–06. European Heart Journal.

[CR26] Heath GW, Parra DC, Sarmiento OL, Andersen LB, Owen N, Goenka S, Montes F, Brownson RC (2012). Evidence-based intervention in physical activity: Lessons from around the world. The Lancet.

[CR27] Hurrelmann K (2009). Social structure and personality development: The individual as a productive processor of reality.

[CR28] ISSP Research Group (2009). International Social Survey Programme: Leisure Time and Sports - ISSP 2007. GESIS Data Archive Cologne.

[CR29] Jekauc D, Reimers AK, Wagner MO, Woll A (2013). Physical activity in sports clubs of children and adolescents in Germany: Results from a nationwide representative survey. Journal of Public Health.

[CR30] Kahn EB, Ramsey LT, Brownson RC, Heath GW, Howze EH, Powell KE, Stone EJ, Rajab MW, Corso P (2002). The effectiveness of interventions to increase physical activity: A systematic review. American Journal of Preventive Medicine.

[CR31] Kahn JA, Huang B, Gillman MW, Field AE, Austin SB, Colditz GA, Frazier AL (2008). Patterns and determinants of physical activity in US adolescents. Journal of Adolescent Health.

[CR32] Karageorghis CI, Vencato MM, Chatzisarantis NL, Carron AV (2005). Development and initial validation of the Brunel lifestyle physical activity questionnaire. British Journal of Sports Medicine.

[CR33] Katzmarzyk PT, Church TS, Craig CL, Bouchard C (2009). Sitting time and mortality from all causes, cardiovascular disease, and cancer. Medicine & Science in Sports & Exercise.

[CR34] Kettner S, Kobel S, Fischbach N, Drenowatz C, Dreyhaupt J, Wirt T, Koch B, Steinacker JM (2013). Objectively determined physical activity levels of primary school children in south-west Germany. BMC Public Health.

[CR35] Klein M, Fröhlich M, Emrich E (2011). Sozialstatus, Sportpartizipation und sportmotorische Leistungsfähigkeit [Social status, sports participation, and motor performance]. Sport und Gesellschaft – Sport and Society.

[CR36] Kuntz B, Frank L, Manz K, Rommel A, Lampert T (2016). Social determinants of swimming ability among children and adolescents in Germany. Results of KiGGS Wave 1. Deutsche Zeitschrift für Sportmedizin.

[CR37] Lampert T, Mensink GBM, Romahn N, Woll A (2007). Körperlich-sportliche Aktivität von Kindern und Jugendlichen in Deutschland. Ergebnisse des Kinder- und Jugendgesundheitssurveys (KiGGS) [Physical activity among children and adolescents in Germany. Results of the german health interview and examination survey for children and adolescents]. Bundesgesundheitsblat–Gesundheitsforschung–Gesundheitsschutz.

[CR38] Lewis LK, Maher C, Belanger K, Tremblay M, Chaput JP, Olds T (2016). At the mercy of the gods: Associations between weather, physical activity, and sedentary time in children. Pediatric Exercise Science.

[CR39] López-Sintas J, Ghahraman A, Pérez Rubiales E (2017). Young people’s leisure patterns: Testing social age, social gender, and linguistic capital hypotheses. Journal of Youth Studies.

[CR40] Loprinzi PD, Trost SG (2010). Parental influences on physical activity behavior in preschool children. Preventive Medicine.

[CR41] Mess F, Woll A (2012). Soziale ungleichheit im kindes- und Jugendalter am Beispiel des Sportengagements in Deutschland [Social inequality in children and adolescents using the example of physical activity in Germany]. Zeitschrift für Soziologie der Erziehung und Sozialisation.

[CR42] Manz K, Schlack R, Poethko-Müller C, Mensink G, Finger J, Lampert T (2014). Körperlich-sportliche Aktivität und Nutzung elektronischer Medien im Kindes- und Jugendalter. Ergebnisse der KiGGS-Studie [Physical activity and electronic media use in children and adolescents]. Bundesgesundheitsblat–Gesundheitsforschung–Gesundheitsschutz.

[CR43] Nagel S, Ehnold P (2007). Soziale Ungleichheit und Beteiligung am Kindersport [Social inequality and participation in children’s sport]. Sportunterricht.

[CR44] Ng M, Fleming T, Robinson M, Thomson B, Graetz N, Margono C (2014). Global, regional, and national prevalence of overweight and obesity in children and adults during 1980–2013: A systematic analysis for the Global Burden of Disease Study 2013. The Lancet.

[CR45] Pugliese J, Tinsley B (2007). Parental socialization of child and adolescent physical activity: A meta-analysis. Journal of Family Psychology.

[CR46] Qi QB, Strizich G, Merchant G, Sotres-Alvarez D, Buelna C, Castaneda SF (2015). Objectively measured sedentary time and cardiometabolic biomarkers in US Hispanic/Latino adults. The hispanic community health study/study of latinos. Circulation.

[CR47] Raudsepp L (2006). The relationship between socio‐economic status, parental support and adolescent physical activity. Acta Paediatrica.

[CR48] Riddoch CJ, Andersen LB, Wedderkopp N, Harro M, Klasson-Heggebo L, Sardinha LB, Cooper AR, Ekelund U (2004). Physical activity levels and patterns of 9- and 15-yr-old European children. Medicine & Science in Sports & Exercise.

[CR49] Robert-Koch-Institut (2013). Referenzperzentile für anthropometrische Maßzahlen und Blutdruck aus der Studie zur Gesundheit von Kindern und Jugendlichen in Deutschland (KiGGS). Berlin.

[CR50] Ruch N, Melzer K, Mäder U (2013). Duration, frequency, and types of children’s activities: Potential of a classification procedure. Journal of Exercise Science & Fitness.

[CR51] Sallis JF, Bauman A, Pratt M (1998). Environmental and policy interventions to promote physical activity. American Journal of Preventive Medicine.

[CR52] Sanders MR, Burke K (2014). The “Hidden” technology of effective parent consultation: A guided participation model for promoting change in families. Journal of Child and Family Studies.

[CR53] Schmiade N, Mutz M (2012). Sportliche eltern, sportliche kinder: Die Sportbeteiligung Von Vorschulkindern im kontext sozialer ungleichheit [Athletic parents, athletic children. Social stratification of preschool children’s participation in sports]. Sportwissenschaft.

[CR54] Schoeppe S, Trost SG (2015). Maternal and paternal support for physical activity and healthy eating in preschool children: A cross-sectional study. BMC Public Health.

[CR55] Steele RM, van Sluijs EMF, Sharp SJ, Landsbaugh JR, Ekelund U, Griffin SJ (2010). An investigation of patterns of children’s sedentary and vigorous physical activity throughout the week. International Journal of Behavioral Nutrition and Physical Activity.

[CR56] Studer F, Schlesinger T, Engel C (2011). Socioeconomic and cultural determinants of sports participation in Switzerland from 2000 to 2008. European Journal for Sport & Society.

[CR57] Sutherland R, Campbell R, Lubans DR, Morgan PJ, Okely AD, Nathan N (2016). ‘Physical activity 4 Everyone’ school-based intervention to prevent decline in adolescent physical activity levels: 12 months (mid-intervention) report on a cluster randomised trial. British Journal of Sports Medicine.

[CR58] Sylvia LG, Bernstein EE, Hubbard JL, Keating L, Anderson EJ (2014). A practical guide to measuring physical activity. Journal of the Academy of Nutrition and Dietetics.

[CR59] Telama R, Yang X, Viikari J, Välimäki I, Wanne O, Raitakari O (2005). Physical activity from childhood to adulthood: A 21-year tracking study. American Journal of Preventive Medicine.

[CR60] Trost SG, Russell RP, Sallis JF, Freedson PS, Taylor WC, Dowda M (2002). Age and gender differences in objectively measured physical activity in youth. Medicine and Science in Sports and Exercise.

[CR61] Trost SG, Sallis JF, Pate RR, Freedson PS, Taylor WC, Dowda M (2003). Evaluating a model of parental influence on youth physical activity. American Journal of Preventive Medicine.

[CR62] Vander Ploeg KA, Maximova K, McGavock J, Davis W, Veugelers P (2014). Do school-based physical activity interventions increase or reduce inequalities in health?. Social Science & Medicine.

[CR63] Verloigne M, Van Lippevelde W, Maes L, Yildirim M, Chinapaw M, Manios Y, Androutsos O, Kovács E, Bringolf-Isler B, Brug J, De Bourdeaudhuij I (2012). Levels of physical activity and sedentary time among 10- to 12-year-old boys and girls across 5 European countries using accelerometers: an observational study within the ENERGY-project. International Journal of Behavioral Nutrition and Physical Activity.

[CR64] Vorwerg Y, Petroff D, Kiess W, Blüher S (2013). Physical activity in 3–6 year old children measured by SenseWear Pro®: Direct accelerometry in the course of the week and relation to weight status, media consumption, and socioeconomic factors. PLoS One.

[CR65] Wang Y, Lobstein T (2006). Worldwide trends in childhood overweight and obesity. International Journal of Pediatric Obesity.

[CR66] Wheeler S (2011). The significance of family culture for sports participation. International Review for the Sociology of Sport.

[CR67] Wijtzes AI, Jansen W, Bouthoorn SH, Pot N, Hofman A, Jaddoe VWV, Raat H (2014). Social inequalities in young children’s sports participation and outdoor play. International Journal of Behavioral Nutrition and Physical Activity.

[CR68] Woll A, Kurth BM, Opper E, Worth A, Bös K (2011). The ‘Motorik-Modul’ (MoMo): physical fitness and physical activity in German children and adolescents. European Journal of Pediatrics.

[CR69] World Health Organization (WHO) (2010). *Global recommendations on physical activity for health*. URL: whqlibdoc.who.int/publications/2010/9789241599979_eng.pdf (27.06.2016).

